# Early Low Protein Diet Aggravates Unbalance between Antioxidant Enzymes Leading to Islet Dysfunction

**DOI:** 10.1371/journal.pone.0006110

**Published:** 2009-07-01

**Authors:** Nicolas Theys, André Clippe, Thomas Bouckenooghe, Brigitte Reusens, Claude Remacle

**Affiliations:** Laboratory of Cell Biology, Institute of Life Sciences, Université catholique de Louvain, Louvain-la-Neuve, Belgium; University of Bremen, Germany

## Abstract

**Background:**

Islets from adult rat possess weak antioxidant defense leading to unbalance between superoxide dismutase (SOD) and hydrogen peroxide-inactivating enzymatic activities, catalase (CAT) and glutathione peroxidase (GPX) rending them susceptible to oxidative stress. We have shown that this vulnerability is influenced by maternal diet during gestation and lactation.

**Methodology/Principal Findings:**

The present study investigated if low antioxidant activity in islets is already observed at birth and if maternal protein restriction influences the development of islet antioxidant defenses. Rats were fed a control diet (C group) or a low protein diet during gestation (LP) or until weaning (LPT), after which offspring received the control diet. We found that antioxidant enzymatic activities varied with age. At birth and after weaning, normal islets possessed an efficient GPX activity. However, the antioxidant capacity decreased thereafter increasing the potential vulnerability to oxidative stress. Maternal protein malnutrition changed the antioxidant enzymatic activities in islets of the progeny. At 3 months, SOD activity was increased in LP and LPT islets with no concomitant activation of CAT and GPX. This unbalance could lead to higher hydrogen peroxide production, which may concur to oxidative stress causing defective insulin gene expression due to modification of critical factors that modulate the insulin promoter. We found indeed that insulin mRNA level was reduced in both groups of malnourished offspring compared to controls. Analyzing the expression of such critical factors, we found that c-Myc expression was strongly increased in islets from both protein-restricted groups compared to controls.

**Conclusion and Significance:**

Modification in antioxidant activity by maternal low protein diet could predispose to pancreatic islet dysfunction later in life and provide new insights to define a molecular mechanism responsible for intrauterine programming of endocrine pancreas.

## Introduction

Oxidative stress (OS) is an important factor in the process of pancreatic β-cell dysfunction which leads to diabetes [1]. Reactive oxygen species (ROS) and/or reactive nitrogen species (RNS) are formed during both pro-inflammatory cytokine-mediated β-cell aggression in Type 1 diabetes mellitus (T1DM) and glucolipotoxicity-mediated β-cell dysfunction in Type 2 diabetes mellitus (T2DM) [1]–[4]. Indeed, there is strong evidence for ROS and RNS participating in the pathogenesis of T1DM while only the participation of ROS can be described as a mechanism of β-cell dysfunction in T2DM [2], [5], [6]. OS features pathological relevance in the appearance of diabetes at adult age because pancreatic islets possess particularly weak antioxidant defense activity [7], [8]. Therefore, the ability of OS to damage pancreatic islets and markedly blunt insulin secretion is not surprising.

The risk of developing metabolic diseases in adulthood is influenced not only by genetic and lifestyle factors but also by environmental factors acting in early life [9]. Epidemiological studies have related that a poor intra-uterine environment may program susceptibility in the progeny to later development of the metabolic syndrome (rev in [10]).

Most of our work to date focused on the influence of a protein restriction (Low Protein, LP) during gestation or gestation and lactation for the offspring. LP diet modified the process of islet cell development leading to impairment in islet cell expansion with a smaller β-cell mass at birth [11]–[14]. When the LP diet was maintained until weaning, this deficiency was more pronounced [11]–[13]. Moreover, maternal LP diet seems to program an increased vulnerability of the endocrine pancreas [15]–[17]. A proteome analysis carried out on *in vitro* neoformed fetal islets showed that ten proteins involved in protein folding and chaperoning were modified, possibly testifying cell stress [18]. Using micro-array analysis, we demonstrated that more than 10% of the gene expression was significantly altered by the maternal low protein diet in fetal islets including antioxidant enzymes modified expression [19]. Furthermore, islets from fetal and adult progeny of dams fed a low protein diet showed a higher rate of apoptosis after cytokines or oxidative stress aggression in *in vitro* experiments [15], [16]. Higher NO° production by islets from adult LP offspring could be an important factor to explain this subsequent cell death [17]. Taken together, these findings suggest that the LP diet increased susceptibility to OS that renders the β-cell more vulnerable and prone to apoptosis.

In addition, the reprogramming of mitochondrial function was proposed as a key adaptation enabling a fetus to survive in a limited environment (rev in [20]). Finally, mitochondrial dysfunction occurs and leads to increased production of ROS and eventually to OS if the defense systems of the cell are overwhelmed [21].

Adult pancreatic islets are equipped with lower antioxidant defenses than other cell types. In the present study, we investigated *(1)* if low antioxidant capacity is already observed at birth by measuring the antioxidant enzyme activity, *(2)* the evolution of these activities during post-natal life. Finally *(3)*, the influence of protein restriction during early life on β-cells antioxidant defense and the control of insulin gene expression were assessed.

## Results

### Litter size and body weight

No difference in litter size was observed between the groups (on average C = 11.9±0.7, LP = 11.7±0.5 pups per litter). The low protein diet caused a significantly smaller mean fetal and newborn body weight as shown in Table 1. The importance of growth retardation was related to the time-window of exposure to the low protein diet. At weaning and at 28 days, LPT offspring was growth restricted compared to C and LP groups, which remained until 3 months of age. Conversely, the body weight gain of the LP progeny was higher compared to C offspring.

**Table 1:** Body weights of fetuses (21.5 days of gestation) and postnatal rats.

	C	LP	LPT
**Fetus**_(n = 73–101)_	5.66±0.06	5.38±0.05**	
**5 days**_(n = 50–78)_	14.8±0.3	14.1±0.2*	11.4±0.2*** †††
**21 days**_(n = 50–78)_	54.5±0.7	54.1±0.8	27.9±0.5*** †††
**28 days**_(n = 28–47)_	88±1.3	87±1.3	57±1.1*** †††
**6 weeks**_(n = 16–29)_	197±4.1	190±3.4	143±4.9*** †††
**3 months**_(n = 16–29)_	381±7.4	417±10*	344±11** †††

Values are means±SEM.

* p<0.05, ^**^p<0.01, ^***^p<0.001 compared with control group.

††† p<0.001 compared to LP group.

### Blood analysis

Table 2 shows fasting blood parameters at 3 months. Compared to C group, insulin level was lower in LPT group whereas the lower value in LP rats did not reach statistical significance. Plasma glucose levels were unaffected in both LP and LPT groups. Offspring of dam fed a low protein diet showed indeed an improved glucose tolerance which is reflected by a significantly lower HOMA-R index. Despite non significant difference in antioxidant potential in plasma of the three groups of rats, the protein nitrosylation was significantly higher in both LP and LPT animals.

**Table 2:** Blood analysis in 3 month-old male rats.

	C	LP	LPT
**Plasma insulin** *(pmol/l)*	141.7±10.7	111.9±7.6	87.4±6.4**
**Plasma glucose** *(mmol/l)*	5.1±0.14	5.1±0.24	5.4±0.35
**Homeostasis model assessment insulin-resistance index** *(HOMA-R)*	4.7±0.4	3.6±0.2*	2.9±0.2**
**Total peroxyl-radical trapping potential** *(µM Trolox equiv.)*	91.4±12.8	87.2±5.5	70.3±4.9
**Nitrotyrosine levels** *(pmol/mg protein)*	0.19±0.02	0.41±0.03**	0.54±0.02**

Values are means±SEM of 6 to 8 animals in each group.

* p<0.05, ^**^p<0.01 *vs* C.

### Antioxidant enzymatic activities in fetal pancreatic islets

**Figure 1:**
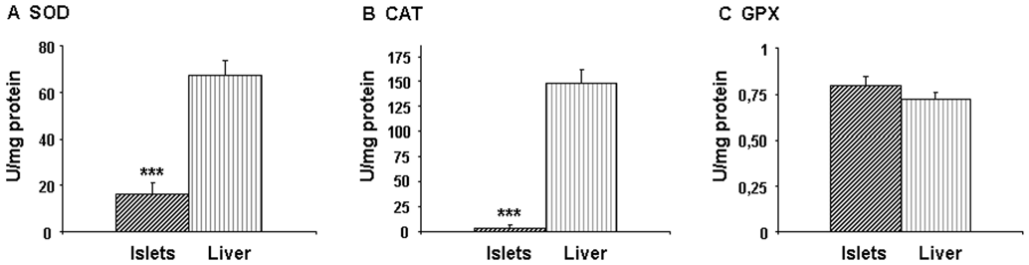
Antioxidant enzyme activity in fetal pancreatic islets and liver. (A) SOD, (B) CAT, (C) GPX. Results are expressed as means±SEM; n = 5–6 independent experiments. ***p<0.001 islets *vs* liver.

Antioxidant activities were measured in islets and compared with those in the liver. Like in previous work [7], [8], the liver was used as a reference tissue since its antioxidant capacity is well documented. As shown in Figure 1, fetal pancreatic islets possessed less SOD and CAT activity than fetal liver. GPX activity was similar in islets and liver at this age.

### Antioxidant enzymatic activities in postnatal pancreatic islets

**Figure 2:**
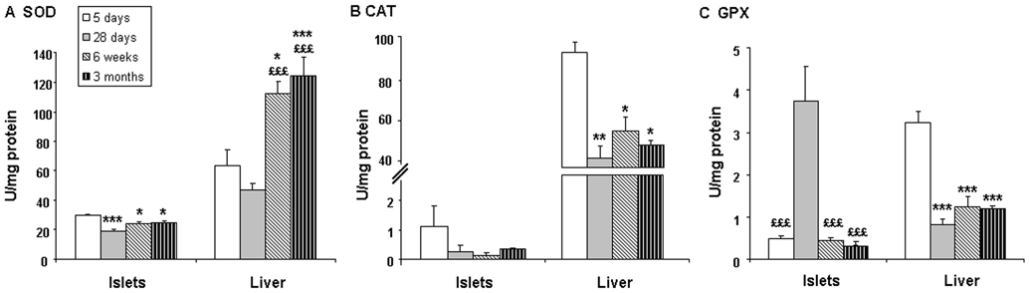
Postnatal evolution of antioxidant enzyme activity in pancreatic islets and liver. (A) SOD, (B) CAT, (C) GPX. Results are expressed as means±SEM; n = 4–7 independent experiments. *p<0.05, **p<0.01, ***p<0.001 *vs* 5 days; £p<0.05, ££p<0.01, £££p<0.001 *vs* 28 days within the same tissue.

Antioxidant enzymatic activity was analyzed in postnatal pancreatic islets isolated at 5 days, 28 days, 6 weeks and 3 months of age. Values obtained for pancreatic islets were compared to those in the liver. Pancreatic islets showed a higher SOD activity at 5 days than at subsequent time points whereas the liver showed an opposite evolution (Figure 2A). Thus, throughout life, islets exhibited a lower SOD activity than liver and this difference was more pronounced with age.

At all ages, CAT activity was very low in islets and we did not observe any significant change from 5 day- to 3 month-old rats (Figure 2B). Compared to liver where the activity was higher in neonate, pancreatic islets had already at birth less than one percent of the liver CAT activity.

Islets exhibited a higher GPX activity at 28 days (Figure 2C), compared to earlier and later ages. At this stage, GPX capacity in islets was significantly higher than in liver at the same age. In the liver, GPX activity was much higher at 5 days than later in life.

### Consequences of a low protein diet on antioxidant enzymatic activities in fetal pancreatic islets

**Figure 3:**
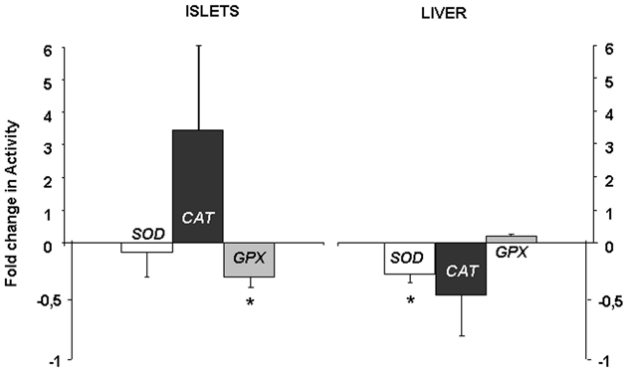
Effect of low protein diet on fetal pancreatic islets and liver antioxidant enzyme activity, SOD (white bars), CAT (black bars), GPX (grey bars). Data are expressed as fold change in activity relative to C; n = 5–6 independent experiments. *p<0.05 *vs* controls.

In islets from fetuses of dams fed a low protein diet during gestation, SOD and CAT activities were unchanged compared to controls, whereas GPX activity was significantly lower (Figure 3). Gene expression corresponding to these enzymes was similar in islets from protein restricted and control fetuses (data not shown). The consequences of low protein diet were different in liver, where CAT and GPX activity was unchanged whereas SOD activity was reduced in LP fetuses compared to controls (Figure 3).

### Consequences of a low protein diet on antioxidant enzymatic activities in postnatal pancreatic islets

**Figure 4:**
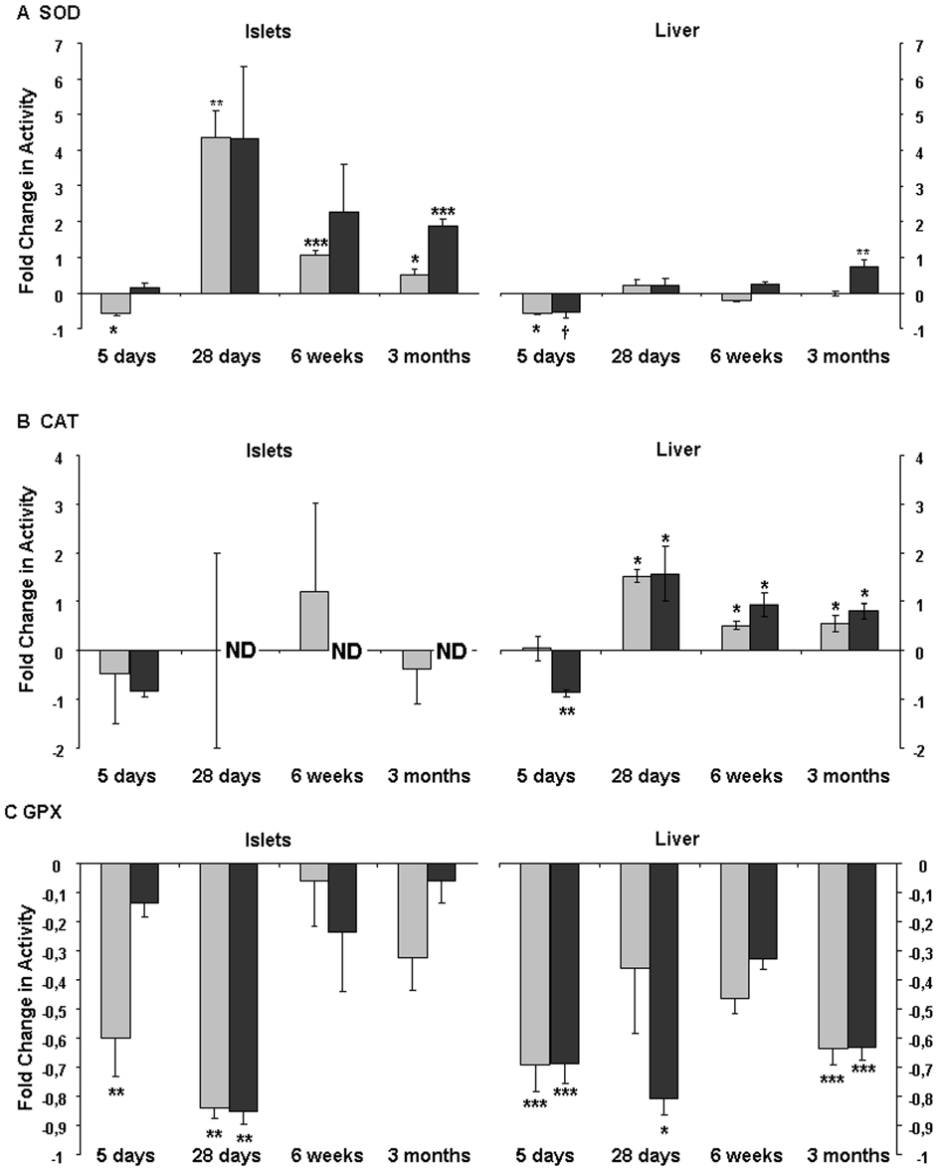
Postnatal effect of low protein diet on antioxidant enzyme activity in pancreatic islets and liver, in LP (grey) and LPT (black) progeny. (A) SOD, (B) CAT, (C) GPX. Data are expressed as fold change in activity relative to C; n = 4–7 independent experiments. *p<0.05, **p<0.01, ***p<0.001, †p = 0.06 *vs* controls.

SOD activity was lower in pancreatic islets from LP newborns (Figure 4A). However, from 28 days, a significant higher SOD activity was measured in islets from LP animals compared to controls. At 3 months, islets obtained from LPT offspring presented a two folds higher SOD activity. In liver, protein restriction led to a lower SOD capacity in LP and LPT pups. No more difference was observed thereafter except at 3 months, when this activity was increased in LPT rats.

Particularly in LPT islets, CAT activity was in a range reaching the detection limit and was so low for many samples that it could not be detected (Figure 4B). Conversely, CAT activity was significantly upregulated in liver from 28 days to 3 months for both LP and LPT offspring.

GPX activity was altered in islets and liver from LP and LPT rats (Figure 4C). It was lower in pancreatic islets from LP newborn while at 28 days, this decline was observed in LPT as well as in LP pancreatic islets. This strong reduction was no more found thereafter. Such lower activity was also detected in liver, particularly in neonates and at 3 months in both protein-restricted progeny compared to controls.

### Consequences of a low protein diet on antioxidant gene expression

**Figure 5:**
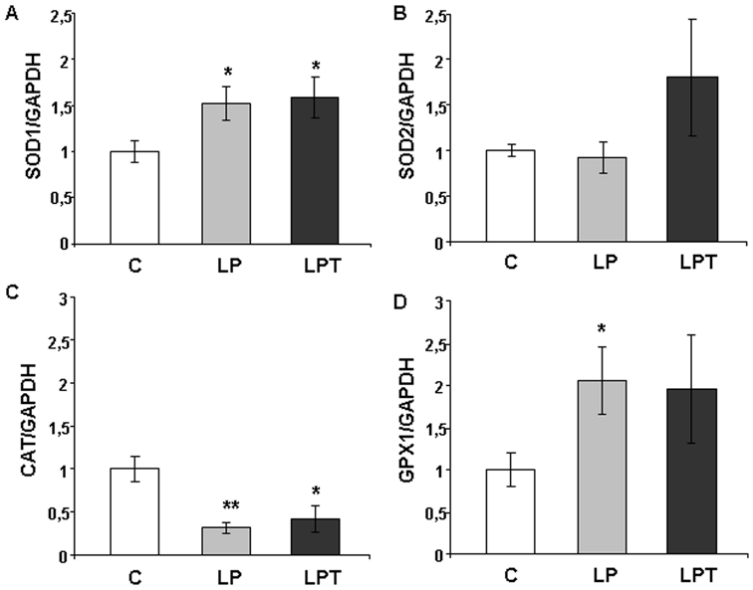
SOD1 (A), SOD2 (B), CAT (C), GPX1 (D) gene expression in pancreatic islets from 3 month-old rats. Results are expressed as means±SEM; n = 6–7 independent experiments. *p<0.05, **p<0.01 *vs* C;

Parallel measurements of antioxidant enzyme mRNA at 3 months enabled to compare expression to activity. A higher SOD1 transcript was observed in islets from 3 month-old low protein progeny whereas SOD2 was slightly increased only in LPT islets (Figure 5A/B). The level of catalase expression was about 30% in islets from LP and LPT animals compared to controls (Figure 5C). GPX1 mRNA was higher in pancreatic islets from LP rats but unchanged in LPT (Figure 5D).

### Consequences of a low protein diet on peroxiredoxin capacity

**Figure 6:**
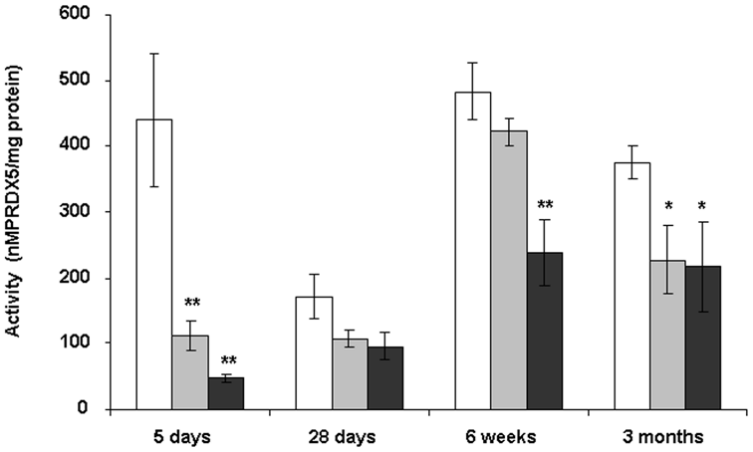
Peroxiredoxin activity in liver from C (white bars), LP (grey bars), LPT (black bars). Results are expressed as means±SEM; n = 4–5 independent experiments. *p<0.05, ** p<0.01 *vs* C;

**Figure 7:**
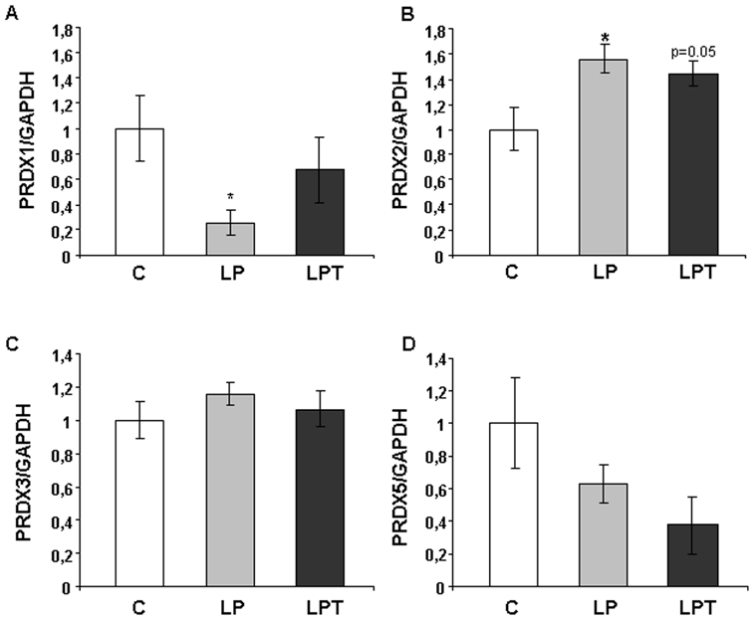
PRDX1 (A), PRDX2 (B), PRDX3 (C) and PRDX5 (D) gene expression in pancreatic islets from 3 month-old rats. Results are expressed as means±SEM; n = 6–7 independent experiments. *p<0.05, **p<0.01 *vs* C;

Peroxiredoxins constitute a less known family of peroxidase. PRDX activity was not detectable in pancreatic islets at any age as well as in the fetal liver. In liver, PRDX activity was lower at birth and at 3 months in LP and LPT animals compared to controls (Figure 6). PRDX1 and PRDX2 gene expression was modified in LP islets (Figure 7A/B) whereas only PRDX2 transcripts tended to be upregulated in LPT islets compared to C. In the liver, PRDX1 mRNA level tended to be reduced in LP (p = 0.07) and was significantly downregulated in LPT rats (p<0.01; Data not shown).

**Figure 8:**
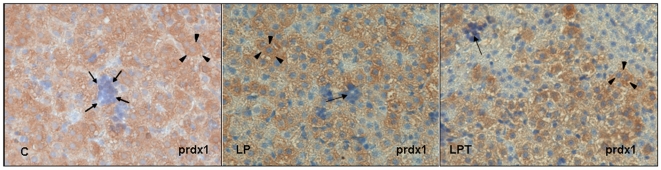
Immunostaining of PRDX1 in liver from 5 day-old newborn control, LP and LPT rats. Hepatocytes (arrows head) appear in brown staining while hematopoietic cells (black arrows) are unstained for PRDX1 for both groups.

Since the liver is a hematopoietic organ during gestation and early postnatal period, we analyzed to which cell type the lower PRDX activity can be attributed. Immunostaining for the different isoforms of peroxiredoxins (1 to 5) was assessed on liver sections from 5 day-old rats and showed no stained hematopoietic cells. Thus, the activity measured in liver from newborn was attributed to hepatocytes. Immunostaining for PRDX1 is presented in Figure 8.

### Consequences of a low protein diet on PDX-1, c-Myc and insulin gene expression

**Figure 9:**
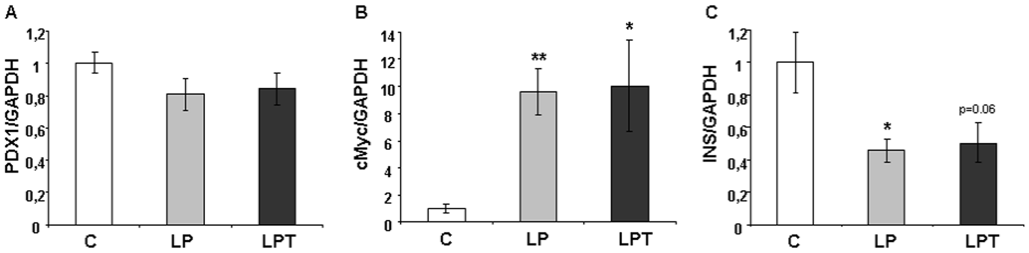
PDX1 (A), cMyc (B) and INS (C) gene expression in pancreatic islets from 3 month-old male rats. Results are expressed as means±SEM; n = 6 independent experiments. *p<0.05, **p<0.01 *vs* C;

Because the level of SOD activity was increased in islets from LP and LPT offspring with no concomitant activation of the hydrogen peroxide-inactivating enzymes CAT and GPX, this unbalance could lead to oxidative stress through a higher hydrogen peroxide production. Exposure of pancreatic islets to oxidative stress causes defective insulin gene expression due to modification of at least two critical factors that modulate the insulin promoter : PDX-1 [22] and c-Myc [23]. Analyzing the expression of these factors (Figure 9), we found that PDX-1 mRNA level was unchanged while c-Myc expression increased strongly in islets from LP and LPT progeny. Insulin mRNA level was reduced in LP and LPT rats compared to controls.

## Discussion

ROS and RNS species are described as contributors of β-cell dysfunction during the development of diabetes [1], [2], [4]. Every cell is equipped with antioxidant defense systems that react with reactive species. Superoxide dismutases (SOD) are the antioxidant enzymes that catalyze the dismutation of the highly reactive superoxide anion to less reactive hydrogen peroxide. Peroxides can be inactivated by catalase (CAT), glutathion peroxidase (GPX) or peroxiredoxin (PRDX). However, pancreatic islets are weakly protected against the toxicity of ROS and RNS as the level of their antioxidant defense has been found to be very low, leading to unbalance between the two antioxidant enzymatic scavenger systems, superoxide dismutases and hydrogen peroxide-inactivating systems, namely CAT and GPX [7], [8], [24]–[26].

The first objective of the present study was to investigate the evolution of the antioxidant activity of pancreatic islets already just before birth until 3 months. The results were compared to antioxidant capacity of the liver, used as a reference tissue [7], [8]. Fetal islets did not feature the unbalanced antioxidant potential described for adult animals. Just before birth, pancreatic islets exhibited a much lower level of SOD and CAT activity than in liver but, a similar GPX activity. Although catalase activity was already negligible in fetal islets, a significant peroxidase capacity through GPX may be protective against the oxidative challenge caused by delivery [27], [28].

Postnatal evolution of antioxidant capacity showed that SOD, CAT and GPX remained particularly weak in pancreatic islets throughout life compared to that in liver. Our findings for adult animals support previous observations comparing the relative values between islets and liver [8]. All antioxidant activities varied with age and the time-course was different regarding the tissue. Weaning is a step where most of the nutritional changes occur and the β-cells have to deal with a change from a high fat diet to a high glucose diet. We showed that in newborn, GPX activity was low and that it increased until 28 days to diminish thereafter. It is known that when GPX activity is experimentally increased to the levels found in other tissues, β-cell are protected from OS [26]. Therefore, the GPX increase may be beneficial for the β-cell before this nutritional transition. In addition, it is known that a postnatal wave of apoptosis occurs during lactation in the normal developing endocrine pancreas [29], [30]. The peak of apoptosis was observed at 2 weeks of age during which 60% of pre-existing β-cells died [31]. Although the functional significance of this remodeling phase is still unknown for β-cells, similar waves of apoptosis which coincide with changing function were described for other cell types [32]. In the pancreas, this wave may prepare the β-cell mass to the nutritional challenge taking place at weaning. Thus, immediately after the remodeling, pancreatic islets acquire a significant intrinsic hydrogen peroxide-inactivating potential which may provide enhanced protection [26], [33].

At each age, pancreatic islets exhibited an extremely weak catalase capacity in the range of 1 or 2% of that in the liver. Catalase overexpresion in cultured insulin producing cells as well as in β-cells from transgenic mice provided protection against oxidative injury [24], [34]. Many authors discussed the relative importance of catalase and other peroxidases in removing H_2_O_2_. Catalase is not expected to intervene in eliminating low levels of H_2_O_2_ but plays an important role in the acquisition of tolerance to oxidative stress [35], [36]. With such a low catalase capacity already at birth, the β-cells should be particularly susceptible to oxidative stress. Indeed, an appropriate level seems to be necessary to challenge high concentration of H_2_O_2_ produced during hyperglycemia or cytokines aggression [24], [26], [34], [37], [38].

Experimental and clinical evidence support the notion that intrauterine malnutrition can contribute to the development of metabolic disease in adult life [9], [10]. Low protein diet impairs the development of fetal pancreas and has long-term consequences on its secretory capacity [11], [14]. Our study investigated a possible mechanism by which the antioxidant unbalance may contribute to the early programming of islets by exposure to a low protein diet. First, we confirmed previous observations showing a significant effect of maternal low protein diet on weight and plasma composition of the offspring [16]. At 3 months, progeny of dams fed a low protein diet during early life presented a lower fasting plasma insulin concentration with a normal fasting glucose level, which may be explained by the improvement of peripheral insulin sensitivity [39], [40]. However, it is known that glucose intolerance occurs later in life in these animals [41], [42].

We demonstrated that GPX activity was significantly reduced in fetal LP islets. This contrasts with the significant GPX activity observed in control fetal islets which is probably important to tolerate oxidative challenge caused by delivery. Islets from fetus of dams fed a low protein diet during gestation are known to be more vulnerable to cytotoxic aggression [15]. Indeed, *in vitro* exposure of fetal islets to a chemical generator of NO° or to cytokines induced apoptosis in β-cell with a significantly greater impact in LP than in C islets [15]. Ten proteins involved in protein folding and chaperoning were found to be altered by the low protein diet in proteomic analysis [18] and data provided by microarray analysis are congruent with an increased sensitivity to oxidative stress in islets from LP fetuses [19]. Moreover, β-cell mass was significantly lower in LP fetuses compared to controls [11]. Although specific targets such as proliferation, vascularization, and growth factors have been identified as involved in the lower β-cell mass in LP progeny [11]–[14], [43], the participation of oxidative stress can not be excluded.

The switch to a normal diet after delivery caused a reduction of islet antioxidant capacity in the newborn, which was not observed when the low protein diet was maintained in LPT pups. Gluckman *et al.* [44] suggested that a mismatch between suboptimal fetal environment and a richer environment really encountered after birth is detrimental. However, in the liver, the duration of exposure to protein restriction did not modify the consequence at 5 days, when both LP and LPT pups presented a lower antioxidant potential than the controls.

In the adult, islets from LP and LPT animals featured a higher SOD activity compared to controls whereas CAT activity was very low. It should be noted that LP offspring recovered a body weight similar to control at weaning whereas LPT offspring remained significantly leaner throughout life. Then, the catch-up growth *per se* in LP animals did not influence the antioxidant potential in islets but, it was shown by others [45], that the reduction in litter size after delivery, 4 pups instead of 8 in our study, to maximize catch-up growth of LP progeny was associated to a lower SOD expression. The same discrepancy is also true for PRDX expression, which shall be discussed below. Those contradictions demonstrate the sensitivity of the enzyme response to nutritional manipulations during the lactating period. In a mouse model, overexpression of MnSOD resulting in overproduction of H_2_O_2_, was found responsible for damaging the β-cells through overriding the limited hydrogen peroxide-inactivating capacity [46]. An increase in SOD activity can be an adaptation to mitochondrial dysfunction in islets from LP and LPT progeny. Using a model of uteroplacental insufficiency, Simmons *et al.* [21] proposed that a reprogramming of mitochondrial function occurred in pancreatic islets as a consequence of a limited energy environment during early life. This reprogramming led to mitochondrial dysfunction which increased production of mitochondrial ROS and MnSOD expression. Modification of mitochondrial function, like a much higher ROS production, was also observed in islets from LP offspring (Theys, unpublished data).

Contrary to the liver where the higher SOD activity in LPT animals was associated to a higher catalase potential, no concomitant increase in any peroxidase potential was observed in islets from LP and LPT rats. Independently of the malnutrition, we know that the vulnerability of normal β-cells is attributed to their unbalance through SOD and hydrogen peroxide-inactivating enzymes [8]. Maternal malnutrition increased this unbalance and probably through that way, enhanced the vulnerability of fetal and adult islets, as reported previously [16], [17].

The method for measuring PRDX activity [47] was not sensitive enough to face the limited quantity of available protein in pancreatic islets. PRDX1 gene expression was lower in LP islets whereas PRDX2 was higher and PRDX-3 and -5 mRNA levels similar in LP and LPT islets compared to C. It should be noted that, as mentioned above, when forced catch-up growth was induced after delivery, offspring of dams fed a LP diet did not present the same modification of PRDX gene expression [45].

Exposure to oxidative stress can cause a decrease in insulin gene expression via loss of the transcription factor PDX-1 [22]. Although PDX-1 mRNA level was unchanged in our study, insulin gene was underexpressed in both LP and LPT islets. Elouil *et al.* [23] showed that H_2_O_2_ reproduced the effect of hyperglycemia on islet mRNA expression of several genes like c-Myc in absence of high glucose concentration. In 3 month-old rats, we observed a strong overexpression of c-Myc in LP and LPT islets compared to controls. It should be noted that high expression of c-Myc in β-cells downregulated insulin gene expression [48] and stimulated apoptosis in rodent β-cells [49]. These modifications may contribute to explain the lower plasma insulin and pancreatic insulin content observed in LP pups [11] as well as the 3 fold-higher apoptotic rate in islets from offspring of dams fed a low protein diet during early life [17].

In conclusion, antioxidant enzymatic capacities of the pancreatic islets of normal rats were very low, already at birth compared to those in the liver. A temporary efficient GPX activity counterbalancing SOD capacity in islets was observed in fetus and after weaning when important modifications in nutritional status occur. Maternal low protein diet altered the islets antioxidant capacity of the progeny. At birth, the changes due to LP diet should render islets more vulnerable to oxidative stress. In adult, we detected an increased unbalance between superoxide-radical-inactivating enzymes and very low hydrogen peroxide-inactivating enzymes, independently of the postnatal continuation of the malnutrition. It is conceivable that those modifications correspond to adaptations to mitochondrial dysfunction. Future studies are in progress to specifically address the role of mitochondria in the consequences of the maternal malnutrition.

## Materials and Methods

### Animals

Adult virgin females Wistar rats (Janvier, Le Genest St Isle, France) were caged overnight with males (four females to one male), and copulation was verified the next morning by detection of spermatozoa in the vaginal smear. Midnight was considered as the time of mating. Pregnant females were then housed individually under controlled conditions (25°C; 14:10 h light:dark cycle) and had free access to their respective diets and to water. Rats were fed either a control diet containing 20% protein (C group) or an isocaloric low protein diet containing 8% protein during gestation only (LP group) or until weaning (LPT group). The composition of these diets has been described previously [11]. At birth (LP group) or after weaning (LPT group), offspring received the control diet. After parturition, all litters were standardized randomly to 8 pups. Results are obtained from male offspring. All procedures were carried out in accordance with “Principles of laboratory animal care” (NIH publication no. 85-23, revised 1985) and with the approval of the animal ethics committee of the Université catholique de Louvain, Belgium.

### Islet collection

Pancreases from about 10 newborns (5 day-old) were first digested with collagenase (Sigma, St. Louis, MO, USA). After washing, the digest was resuspended in HBSS (Hank's Balanced Salt Solution, pH 7.4) containing 1% foetal bovine serum (Gibco, Paisley, UK). For the other ages (28 days, 6 weeks and 3 months), after obstruction of the junction of the common bile duct with duodenum, a catheter was introduced into the bile duct close to the liver. Collagenase P (Roche, Mannhein, Germany) was injected into the duct to distend the pancreas. The pancreas containing collagenase was laid down into a tube and placed in 37°C water bath to allow digestion of the exocrine tissue. After washing, islets were isolated by hand picking and immediately homogenised in PBS-1% Triton buffer. Islets from 21.5 day-old foetuses (F21.5) were obtained after 7 days of culture as described previously [18].

### Liver collection

Livers were removed rapidly after decapitation, washed in HBSS to remove contaminating blood, frozen and stored at −80°C until utilized. Frozen tissues were homogenized as described previously [50].

### Fixation and tissue processing for immunohistochemistry

Livers from 5 day-old rats were fixed in 0.2% glutaraldehyde −2% paraformaldehyde in PBS 0.1 M, dehydrated and embedded in paraffin. Tissue sections (7 µM) were collected on poly-L-lysine-coated glass slides.

### Immunohistochemistry

Tissue sections were submitted to a 12-min microwave treatment in citrate buffer to induced epitope retrieval and incubated 30 min with a blocking buffer (3% casein in Tris-buffered saline) before a 4°C overnight incubation with the primary antibodies. Second antibodies were incubated for 1 h at room temperature and revealed by diaminobenzidine (Vector Laboratories, Compiegne, France). Primary antibodies were rabbit polyclonal anti-peroxiredoxins (1 to 5) antibodies and were used at 1∶1000 dilution (Autogen Bioclear, Wiltshire, UK). Secondary antibodies were pig anti-rabbit coupled to peroxidase and used at 1∶1000. Controls were made by omitting the primary antibody.

### Antioxidant enzyme activities

Superoxide dismutase (SOD) activity was measured using the chemiluminescence of imidazolopyrazinone molecule (mCLZm) when this reacts with O_2_^−^. This method was adapted from Jansens et al. [51] allowing to detect very low activity in samples. Exploiting the mCLZm chemiluminescence property, total SOD activity was estimated by the inhibition of light production in conditions where the enzymatically generated O_2_^−^ was the rate limiting factor of the luminescence. In these circumstances, SOD competed with mCLZm for O_2_^−^, and the luminescence was proportional to SOD activity in the sample. The injection of 50 µl of an hypoxanthine solution (34 mg ml^−1^) into a mixture composed of 25 µl of diluted sample, 165 µl of a 100 mM phosphate buffer (pH 7.8) containing 0.6 mM EDTA and 0.01% bovine serum albumin (BSA), 5 µl 0.8 µM mCLZm and 5 µl xanthine oxidase (34.5 mU ml^−1^) initiated the light emission. SOD activity was calculated using a standard curve established with SOD1 purified from bovine erythrocytes (Sigma-Aldrich, Bornem, Belgium) in PBS-1% Triton buffer.

Catalase (CAT) activity measurement was carried out using a microplate luminometer (Berthold, Oak Ridge, TN, USA) equipped with two 50 µl injectors. A first injection of a 10 µM H_2_O_2_ solution initiated its reduction by the CAT contained in the diluted sample, which was added to a 100 µl phosphate buffer 100 mM (pH 7.8) containing 0.6 mM EDTA. After 30 minutes of incubation, the injection of a solution containing 20 mM luminol and 11.6 mU ml^−1^ horseradish peroxydase produced an emission of light whose intensity was proportional to the H_2_O_2_ remaining in the mixture. Catalase activity in the sample was obtained using standard curve established with purified bovine liver catalase (Sigma-Aldrich) solubilized in the homogenization buffer.

Glutathione peroxidase (GPX) activity was measured according to the spectrophotometric method of Paglia and Valentine [52]. Diluted samples (15 µl) were added to 115 µl of a 50 mM Tris-HCl buffer (pH 7.6) containing 0.1 mM EDTA, 0.14 mM NADPH, 1 mM GSH and 1 U ml^−1^ glutathione reductase. The consumption of NADPH was monitored at 340 nm after the addition of 0.2 mM tert-butyl hydroperoxide (15 µl) in a spectrophotometer (SpectraMax 190, Molecular Devices, Sunnyvale, USA). GPX activity was estimated from a standard construct with GPX purified from bovine erythrocytes.

Thioredoxin peroxidase activity was assayed following the technique of Kim et al. [47] using recombinant (rec) yeast (y) thioredoxin/thioredoxin reductase system. Both enzymes were produced in E.Coli. Briefly, the spectrophotometric assay was carried out in a 100 µl reaction mixture containing PBS 0.1 M, 500 µM NADPH, 4 µM *rec* yTXN, 2 µM *rec* yTXNRD, and sample. The reaction was initiated by adding H_2_O_2_ at the final concentration of 33 µM. NADPH oxidation was monitored by following absorbance at 340 nm for 30 min at 37°C. Thioredoxin peroxidase activity was estimated from a standard construct with recombinant PRDX5 and expressed in equivalent nM PRDX5.

### Glucose and insulin measurements

Blood samples were collected from 3 month-old rats in tubes containing heparin and used for preparation of plasma. For measurement of glucose concentration, 50 µl of blood were added to 500 µl HClO_4_ (0.33 mol/l) for protein precipitation. Plasma glucose concentration was determined by the glucose oxidase colorimetric method (Stanbio, Boerne, TX, USA). Plasma insulin concentration was measured by ELISA test using the Mercodia Ultrasensitive Rat Insulin ELISA Kit (Uppsala, Sweden). Insulin resistance was calculated by a homeostasis model assessment (HOMA-R) = [fasting serum insulin (mU/l) times fasting blood glucose (mmol/l)]/22.5.

### Plasma oxidative markers

Blood samples were collected from 3 month-old rats in tubes containing EDTA, used for preparation of plasma. Plasma samples were stored at −80°C until analysis. Plasma nitrotyrosine levels were assessed with a commercial ELISA kit (HyCult Biotechnology, Uden, Holland).

### Plasma total peroxyl radical antioxidant trapping potential (TRAP)

This measure is based on the protection afforded by plasma antioxidants against the decay of luminol luminescence emission during a controlled peroxidation reaction initiated by AAPH (2-2′-Azobis(2-methylpropionamidine)dihydrochloride). Reaction mixture (100 µl) contained plasma diluted in phosphate buffer (100 µM, pH 7.4) was laid down on 96 well plate. A luminometer (Berthold) equipped with two injectors added successively 50 µl luminol (20 mM) and AAPH (6 mM). The decay of luminol luminescence was monitored every 30 sec during 30 min. The results were standardized using Trolox (Sigma-Aldrich) as a reference. TRAP values were calculated from the length of the lag-phase due to the sample compared with that of Trolox and were expressed as µM of Trolox equivalent.

### Real-Time RT-PCR

The level of mRNA expression was measured by real-time reverse transcription (RT)-PCR. Total RNA was extracted from islets/liver with the NucleoSpin® RNAII/L (Macherey-Nagel, Hoerdt, France). All RNA used for quantitative real-time gene analysis met the minimum requirement of at least a 1.8 ratio of 18S∶28S rRNA. The RNA aliquots were stored at −80°C before use. First-strand cDNAs were synthesised from 1 µg DNA-free total RNA by RT using random hexamers and SuperScript III enzyme (InVitrogen, Paisley, UK). The template concentration per reaction represented one tenth of the cDNA reaction performed on 1 µg total RNA. Amplification was achieved in 20 µl reaction mixture containing 2 µl cDNA, 25 pmol/l of each oligonucleotide primer (Sigma-Aldrich, Bornem, Belgium) and 2× Sybr Green Master Mix (Eurogentec, Seraing, Belgium). Oligonucleotides are presented in Table 3. After activation of the hot-start DNA polymerase for 10 min at 95°C, solutions underwent 40 cycles of amplification in a sequence detection system (ABI PRISM 7000; Applied Biosystems, Lennik, Belgium). Amplification parameters included 15 s denaturation at 94°C and a 1 min annealing and extension step at 60°C. Direct detection of PCR products was monitored by measuring the increase in fluorescence caused by the binding of SYBR Green to double-stranded DNA. RT blank control PCRs showed no product amplification for all genes examined in this study. The level of mRNA expression was calculated using the threshold cycle (C_t_) value, the number of PCR cycles at which the fluorescence signal during the PCR reaches a fixed threshold. For each sample, the C_t_ both for the gene of interest and for the housekeeping gene GAPDH were determined to calculate ΔC_t,sample_ (C_t, target gene_−C_t, housekeeping gene_), thus normalizing the data and correcting for differences in amount and/or quality between the different samples. The expression level was reported to a calibrator consisting of cDNA from control rats. Subsequently, ΔΔC_t_ (ΔC_t,sample_−ΔC_t,calibrator_) was determined, and the relative expression levels were calculated from 2^−ΔΔCt^ according to the manufacturer's instructions (Applied Biosystems, Foster City, CA, USA). Messenger RNA expression levels are thus indicated as arbitrary units±SEM.

**Table 3:** Sequences of oligonucleotide primers and Genbank accession number.

Gene	5′-Sense Primer-3′	5′-Antisense Primer-3′	Genbank
***SOD1***	AGA GGC ATG TTG GAG ACC TGG	CGG CCA ATG ATG GAA TGC T	NM_017050
***SOD2***	AGC AAG GTC GCT TAC AGA TTG C	CAG TGG AAT AAG GCC TGT GGT T	Y00497
***CAT***	CAT GAA TGG CTA TGG CTC ACA C	CAA CAG GCA AGT TTT TGA TGC C	NM_012520
***GPX1***	AGA AGT GCG AGG TGA ATG GTG A	TTC CAG GAA ATG TCG TTG CG	NM_030826
***PRDX1***	GCT CAC GGT TGG TTC TGT TTG	TGA ATT GTC CAT CCG GCA T	NM_057114
***PRDX2***	TCC TTC GCC AGA TCA CAG TCA	CCT TGC TGT CAT CCA CAT TGG	NM_017169
***PRDX3***	TTC TCA TGC CAA AAG AGA GCC	ACA AAG CCC ATG GAG CAG TAC	NM_022540
***PRDX5***	GAA AGG AGC AGG TTG GGA GTG T	CCC AGG GAC TCC AAA CAA AA	NM_053610
***PDX1***	TTT CCC GAA TGG AAC CGA GA	GCG TGA GCT TTG GTG GAT TTC	NM_022852
***cMyc***	GGG ATC CTG AGT CGC AGT ATA AAA	GTC AGA AAA AAA CGC CCG AA	M18819
***INS I &II***	GCC CAG GCT TTT GTC AAA CA	AAA CCA CGT TCC CCA CAC A	NM_019129 NM_019130
***GAPDH***	TGA CTC TAC CCA CGG CAA GTT	CTT CCC ATT CTC AGC CTT GAC T	NM_017008

SOD1 or Cu/ZnSOD: Copper/Zinc dismutase; SOD2 or MnSOD: managanese superoxide dismutase; CAT: catalase; GPX1: Glutathione peroxidase 1; PRDX1: peroxiredoxin 1; PRDX2: peroxiredoxin 2; PRDX3: peroxiredoxin 3; PRDX5: peroxiredoxin 5; PDX1 : pancreatic and duodenal homeobox-1; cMyc : cellular myelocytomatosis oncogene; INS : insulin; GAPDH : glyceraldehyde-3-phosphate dehydrogenase.

### Statistical analysis

Results were reported as means±SEM. Statistical analyses were performed using one or two-way ANOVA followed by Tukey or Bonferroni post-tests. A *p* value of less than 0.05 was considered as statistically significant.
